# On Force

**Published:** 1863-07

**Authors:** 


### SELECTED.

### ON FORCE.

From a Lecture by Prof. TYNDALL, before the Royal Institution.

We all have ideas more or less distinct regarding force; we know in a general way what muscular force means, and each of us would less willingly accept a blow from a pugilist than have his ears boxed by a lady. But these general ideas are not now sufficient for us; we must learn how to express numerically the exact mechanical value of the two blows; this is the first point to be cleared up.

A sphere of lead weighing 1 lb. was suspended at a height of 16 feet above the theatre floor. It was liberated, and fell by gravity. That weight required exactly a second to fall to the earth from that elevation; and the instant before it touched the earth, it had a velocity of 32 feet a second. That is to say, if at that instant the earth were annihilated, and its attraction annulled, the weight would proceed through space at the uniform velocity of 32 feet a second.

Suppose that instead of being pulled downward by gravity, the weight is cast upward in opposition to the force of gravity, with what velocity must it start from the earth's surface in order to reach a height of 16 feet? With a velocity of 32 feet a second. This velocity imparted to the weight by the human arm, or by any other mechanical means, would carry the weight up to the precise height from which it has fallen.

Now, the lifting of the weight may be regarded as so much mechanical work. I might place a ladder against the wall, and carry the weight up a height of 16 feet; or I might draw it up to this height by means of a string and pulley, or I might suddenly jerk it up to a height of 16 feet. The amount of work done in all these cases, as far as the raising of the weight is concerned, would be absolutely the same. The absolute amount of work done depends solely upon two things: first of all, on the quantity of matter that is lifted; and secondly, on the height to which it is lifted. If you call the quantity or mass of matter $m$, and the height through which it is lifted $h$, then the product of $m$ into $h$, or $mh$, expresses the amount of work done.

Supposing now, that instead of imparting a velocity of 32 feet a second to the weight we impart twice this speed, or 64 feet a second. To what height will the weight rise? You might be disposed to answer, "To twice the height;" but this would be quite incorrect. Both theory and experiment inform us that the weight would rise to four times the height; instead of twice 16, or 32 feet, it would reach four times 16, or 64 feet. So also, if we treble the starting velocity, the weight would reach nine times the height; if we quadruple the speed at starting, we attain sixteen times the height. Thus, with a velocity of 128 feet a second at starting, the weight would attain an elevation of 256 feet. Supposing we augment the velocity of starting seven times, we should raise the weight to 49 times the height, or to an elevation of 784 feet.

Now the work done—or, as it is sometimes called, the *mechanical effect*—as before explained, is proportional to the height, and as a double velocity gives four times the height, a treble velocity nine times the height, and so on, it is perfectly plain that the mechanical effect increases as the square of the velocity. If the mass of the body be represented by the letter $m$, and its velocity by $v$, then the mechanical effect would be represented by $m v^2$. In the case considered, I have supposed the weight to be cast upward, being opposed in its upward flight by the resistance of gravity; but the same holds true if I send the projectile into water, mud, earth, timber, or other resisting material. If, for example, you double the velocity of a cannon-ball, you quadruple its mechanical effect. Hence the importance of augmenting the velocity of a projectile, and hence the philosophy of Sir William Armstrong in using a 50 lb. charge of powder in his recent striking experiments.

The measure then of mechanical effect is the mass of the body multiplied by the square of its velocity.

Now, in firing a ball against a target, the projectile, after collision, is often found hissing hot. Mr. Fairbairn informs me that in the experiments at Shœburyness it is a common thing to see a flash of light, even in broad day, when the ball strik s the target. And if I examine my lead weight after it has fallen from a height I also find it heated. Now, here experiment and reasoning lead us to the remarkable law that the amount of heat generated, like the mechanical effect, is proportional to the product of the mass into the square of the velocity. Double your mass, other things being equal, and you double your amount of heat; double your velocity, other things remaining equal, and you quadruple your amount of heat. Here then we have common mechanical motion destroyed and heat produced. I take this violin bow and draw it across this string. You hear the sound. That sound is due to motion imparted to the air, and to produce that motion a certain portion of the muscular force of my arm must be expended. We may here correctly say, that the mechanical force of my arm is converted into music. And in a similar way we say that the impeded motion of our descending weight, or of the arrested cannon ball, is converted into heat. The mode of motion changes, but it still continues motion; the motion of the mass is converted into a motion of the atoms of the mass; and these small motions communicated to the nerves, produce the sensation which we call heat. We, moreover, know the amount of heat which a given amount of mechanical force can develop. Our lead ball, for example, in falling to the earth, generated a quantity of heat sufficient to raise the temperature of its own mass three-fifths of a Fahrenheit degree. It reached the earth with a velocity of 32 feet a second, and forty times this velocity would be a small one for a rifle bullet; multiplying three-fifths by the square of 40, we find that the amount of heat developed by a collision with the target would, if wholly concentrated in the lead, raise its temperature 960 degrees. This would be more than sufficient to fuse the lead. In reality, however, the heat developed is divided between the lead and the body against which it strikes; nevertheless, it would be worth while to pay attention to this point and to ascertain whether rifle-bullets do not, under some circumstances, show signs of fusion.

From the motion of sensible masses, by gravity and other means, the speaker passed to the motion of atoms towards each other by chemical affinity. A collodion balloon filled with a mixture of chlorine and hydrogin was hung in the focus of a parabolic mirror, and in the focus of a second mirror 20 ft. distant a strong electric light was suddenly generated; the instant the light fell upon the balloon the atoms within it fell together with explosion, and hydrochloric acid was the result. The burning of charcoal in oxygen was an old experiment, but it had now a significance beyond what it used to have; we now regard the act of combination on the part of the atoms of oxygen and coal exactly as we regard the clashing of a falling weight against the earth. And the heat produced in both cases is referrable to a common cause. This glowing diamond, which burns in oxygen as a star of white light, glows and burns in consequence of the falling of atoms of oxygen against it. And could we measure the velocity of the atoms when they clash, and could we find their number and weight, multiplying the mass of each atom by the square of its velocity, and adding all together, we should get a number representing the exact amount of heat developed by the union of the oxygen and carbon.

Thus far, we have regarded the heat developed by the clashing of sensible masses and of atoms. Work is expended in giving motion to these atoms or masses, and heat is developed. But we reverse this process daily, and by the expenditure of heat, execute work. We can raise a weight by heat; and in this agent we possess an enormous store of mechanical power. This pound of coal, which I hold in my hand, produces by its combination with oxygen an amount of heat which, if mechanically applied, would suffice to raise a weight of 100 lbs. to a heiuht of 20 miles above the earth's surface. Conversely, 100 lbs. falling from a height of 20 miles, and striking against the earth, would generate an amount of heat equal to that developed by the combustion of a pound of coal. Wherever work is done by heat, heat disappears. A gun which fires a ball is less heated than one which fires blank cartridge. The quantity of heat communicated to the boiler of a working steam-engine is greater than could be obtained from the re-condensation of the steam after it had done its work; and the amount of work performed is the exact equivalent of the amount of heat lost. Mr. Smyth informed us in his interesting course, that we dig annually 84 millions of tons of coal from our pits. The amount of mechanical force represented by this quantity of coal seems perfectly fabulous. The combustion of a single pound of coal, supposing it to take place in a minute, would be equivalent to the work of 300 horses; and if we suppose 108 millions of horses working day and night, with unimpaired strength, for a year, their united energies would enable them to perform an amount of work just equivalent to that which the annual produce of our coal-fields would be able to accomplish.

Comparing the energy of the force with which oxygen and carbon unite together, with ordinary gravity the chemical affinity seems almost infinite. But let us give gravity fair play; let us permit it to act throughout its entire range. Place a body at such a distance from the earth that the attraction of the earth is barely sensible, and let it fall to the earth from this distance. It would reach the earth with a final velocity of 36,747 feet in a second; and on collision with the earth the body would generate about twice the amount of heat generated by the combustion of an equal weight of coal. We have stated that by falling through a space of 16 feet our lead bullet would be heated three-fifths of a degree; but a body falling from an infinite distance has already used up 1,299,990 parts out of 1,300,000 of the earth's pulling power, when it has arrived within 16 feet of the surface; on this space only 1,130,000ths of the whole force is exerted.

Let us now turn our thoughts for a moment from the earth towards the sun. The researches of Sir John Herschel and M. Pouillet have informed us of the annual expenditure of the sun as regards heat; and by an easy calculation we ascertain the precise amount of the expenditure which falls to the share of our planet. Out of 2,200 million parts of light and heat the earth receives one. The whole heat emitted by the sun id a minute would be competent to boil 12,000 millions of ice-cold water. How is this enormous loss made good? Whence is the sun's heat derived, and by what means is it maintained? No combustion, no chemical affinity with which we are acquainted would be competent to produce the temperature of the sun's surface. Besides, were the sun a burning body merely, its light and heat would assuredly speedily come to an end. Supposing it to be a solid globe of coal, its combustion would only cover 4,600 years of expenditure. In this short time it would burn itself out. What agency can then produce the temperature and maintain the outlay? We have already regarded the case of a body falling from a great distance towards the earth, and found that the heat generated by its collision, would be twice that produced by the combustion of an equal weight of coal. How much greater must be the heat developed by a body falling towards the sun! The maximum velocity with which a body can strike the earth is about 7 miles in a second; the maximum velocity with which it can strike the sun is 390 miles in a second. And as the heat developed by the collision is proportional to the square of the velocity destroyed, an asteroid falling into the sun with the above velocity would generate about 10,000 times the quantity of heat generated by the combustion of an asteroid of coal of the same weight. Have we any reason to believe that such bodies exist in space, and that they may be raining down upon the sun? The meteorites flashing through the air are small planetary bodies, drawn by the earth's attraction, and entering our atmosphere with planetary velocity. By friction against the air they are raised to incandescence and caused to emit light and heat. At certain seasons of the year they shower down upon us in great numbers. In Boston 240,000 of them were observed in nine hours. There is no reason to suppose that the planetary system is limited to "vast masses of enormous weight;" there is every reason to believe that space is stocked with smaller masses, which obey the same laws as the large ones. That lenticular envelope which surrounds the sun, and which is known to astronomers as the Zodiacal light, is probably a cloud of meteors; and moving as they do in a resisting medium they must continually approach the sun. Falling into it, they would be competent to produce the heat observed, and this would constitute a source from which the annual loss of heat would be made good. The sun, according to this hypothesis, would be continually growing larger; but how much larger? Were our moon to fall into the sun it would develop an amount of heat sufficient to cover one or two years' loss; and were our earth to fall into the sun a century's loss would be made good. Still, our moon and our earth, if distributed over the surface of the sun, would utterly vanish from perception. Indeed, the quantity of matter competent to produce the necessary effect would, during the range of history, produce no appreciable augmentation in the sun's magnitude. The augmentation in the sun's attractive force would be more appreciable. However this hypothesis may fare as a representant of what is going on in nature, it certainly shows how a sun might be formed and maintained by the application of known thermodynamic principles.

Our earth moves in its orbit with a velocity of 68,040 miles an hour. Were this motion stopped, an amount of heat would be developed sufficient to raise the temperature of a globe of lead of the same size as the earth 384,000° of the centigrade thermometer. It has been prophecied that "the elements shall melt with fervent heat." The earth's own motion embraces the condition of fulfilment; stop that motion, and the greater part, if not the whole of her mass, would be reduced to vapor. If the earth fell into the sun, the amount of heat developed by the shock would be equal to that developed by the combustion of 6435 earths of solid coal.

There is one other consideration connected with the permanence of our present terrestrial conditions, which is well worthy of our attention. Standing upon one of the London bridges, we observe the current of the Thames reversed, and the water poured upward twice a-day. The water thus moved rubs against the river's bed and sides, and heat is the consequence of this friction. The heat thus generated is in part radiated into space, and then lost, as far as the earth is concerned. What is it that supplies this incessant loss? The earth's rotation. Let us look a little more closely at the matter. Imagine the moon fixed, and the earth turning like a wheel from west to east in its diurnal rotation. Suppose a high mountain on the earth's surface; on approaching the moon's meridian, that mountain is, as it were, laid hold of by the moon, and forms a kind of handle by which the earth is pulled more quickly round. But when the meridian is passed the pull of the moon on the mountain would be in the opposite direction, it now tends to diminish the velocity of rotation as much as it previously augmented it; and thus the action of all fixed bodies on the earth's surface is neutralized. But suppose the mountain to lie always to the east of the moon's meridian, the pull then would be always exerted against the earth's rotation, the velocity of which would be diminished in a degree corresponding to the strength of the pull. The tidal wave occupies this position—it lies always to the east of the moon's meridian, and thus the waters of the ocean are in part dragged as a brake along the surface of the earth; and as a brake they must diminish the velocity of the earth rotation. The diminution, though inevitable, is, however, too small to make itself felt within the period over which observations on the subject extend. Supposing, then, that we turn a mill by the action of the tide, and produce heat by the friction of the millstones; that heat has an origin totally different from the heat produced by another mill which is turned by a mountain stream. The former is produced at the expense of the earth's rotation; the latter at the expense of the sun's radiation.

The sun, by the act of vaporization lifts mechanically all the moisture of our air. It condenses and falls in the form of rain,—it freezes and falls as snow. In this solid form it is piled upon the Alpine heights, and furnishes materials for the glaciers of the Alps. But the sun again interposes, liberates the solidified liquid, and permits it to roll by gravity to the sea. The mechanical force of every river in the world, as it rolls towards the ocean it draws from the heat of the sun. No streamlet glides to a lower level without having been first lifted to the elevation from which it springs by the mighty power of the sun. The energy of winds is also due entirely to the sun; but there is still another work which he performs, and his connection with which is not so obvious. Trees and vegetables grow upon the earth, and when burned they give rise to heat, and hence to mechanical energy. Whence is this power derived? You see this oxide of iron, produced by the falling together of the atoms of iron and oxygen; here also is a transparent gas which you cannot now see—carbonic acid gas—which is formed by the falling together of carbon and oxygen. These atoms thus in close union resemble our lead weight while resting on the earth; but I can wind up the weight and preserve it for another fall; and to these atoms can be wound up, separated from each other, and thus enabled to repeat the proceas of combination. In the building of plants carbonic acid is the material from which the carbon of the plant is derived; and the solar beam is the agent which tears the atoms asunder, setting the oxygen free, and allowing the carbon to aggregate in woody fibre. Let the solar rays fall upon a surface of sand; the sand is heated, and finally radiates away as much heat as it receives; let the same beams fall upon a forest, the quantity of heat given back is less than the forest receives, for the energy of a portion of the sun beams is invested in building up the trees in the manner indicated. Without the sun the reduction of the carbonic acid cannot be effected, and an amount of sunlight is consumed exactly equivalent to the molecular work done. Thus trees are formed; thus the cotton on which Mr. Bazley discoursed last Friday is formed. I ignite this cotton, and it flames; the oxygen again unites with its beloved carbon; but an amount of heat equal to that which you see produced by its combustion was sacrificed by the sun to form that bit of cotton.

But we cannot stop at vegetable life, for this is the source, mediate or immediate, of all animal life. The sun severs the carbon from its oxygen; the animal consumes the vegetable thus formed, and in its arteries a reunion of the severed elements take place, and produce animal heat. Thus, strictly speaking, the process of building a vegetable is one of winding up; the process of building an animal is one of running down. The warmth of our bodies, and every mechanical energy which we exert, trace their lineage directly to the sun. The fight of a pair of pugilists, the motion of an army, or the lifting of his own body up mountain slopes by an Alpine climber, are all cases of mechanical energy drawn from the sun. Not, therefore, in a poetical, but in a purely mechanical sense, are we the children of the sun. Without food we should soon oxidise our own bodies. A man weighing 150 lbs. has 64 lbs. of muscle; but these, when diried, reduce themselves to 15 lbs. During an ordinary day's work, for eighty days, this mass of muscle would be wholly oxidised. Special organs which do more work would be more quickly oxidized: the heart, for example, if entirely unsustained, would be oxidized in about a week. Take the amount of heat due to the direct oxidation of a given amount of food; a less amount of heat is developed by this food in the working animal frame, and the missing quantity is the exact equivalent of the mechanical work which the body accomplishes.

I might extend these considerations; the work, indeed, is done to my hand—but I am warned that I have kept you already too long. To whom, then, are we indebted for the striking generalisations of this evening's discourse? All that I have laid before you is the work of a man of whom you have scarcely ever heard. All that I have brought before you has been taken from the labors of a German physician, named Mayer. Without external stimulus, and pursuing his profession as town physician in Heilbronn, this man was the first to reduce the conception of the interaction of natural forces to clearness in his own mind. And yet he is scarcely ever heard of in scientific lectures, and even to scientific men his merits are but partially known. Led by his own beautiful researched, and quite independent of Mayer, Mr. Joule published his first paper on the "Mechanical Value of Heat," in 1843; but in 1842 Mayer had actually calculated the mechanical equivalent of heat from data which a man of rare originality alone could turn to account. From the velocity of sound in air Mayer determined the mechanical equivalent of heat. In 1854 he published his Memoir on "Organic Motion," and applied the mechanical theory of heat in the most fearless and precise manner to vital processes. He also embraced the other natural agents in his chain of conservation. In 1853, Mr. Waterston proposed, independently, the meteoric theory of the sun's heat, and in 1854, Professor William Thomson applied his admirable mathematical powers to the development of the theory; but six years previously the subject had been handled in a masterly manner by Mayer, and all that I have said on this subject has been derived from him. When we consider the circumstances of Mayer's life, and the period at which he wrote, we cannot fail to be struck with astonishment at what he has accomplished. Here was a man of genius working in silence, animated solely by a love of his subject, and arriving at the most important results some time in advance of those whose lives were entirely devoted to Natural Philosophy. It was the accident of bleeding a feverish patient, in Java, in 1840, that led Mayer to speculate on these subjects. He noticed that the venous blood in the tropics was of a much brighter red than in colder latitudes, and his reasoning on this fact led him into the laboratory of natural forces, where he has worked with such signal ability and success. Well, you will desire to know what has become of this man. His mind gave way; he became insane, and he was sent to a lunatic asylum. In a Biographical Dictionary of his country it is stated that he died there; but this is incorrect. He recovered; and, I believe, is at this moment a cultivator of vineyards in Heilbronn.—*Chemical News.*

